# Understanding and reporting odds ratios as rate-ratio estimates in case-control studies

**DOI:** 10.7189/jogh.13.04101

**Published:** 2023-09-15

**Authors:** Steven Kerr, Sander Greenland, Karen Jeffrey, Tristan Millington, Stuart Bedston, Lewis Ritchie, Colin R Simpson, Adeniyi Francis Fagbamigbe, Amanj Kurdi, Chris Robertson, Aziz Sheikh, Igor Rudan

**Affiliations:** 1 Centre for Medical Informatics, Usher Institute, The University of Edinburgh, Edinburgh, Scotland, UK; 2 Department of Epidemiology and Department of Statistics, University of California, Los Angeles, California, USA; 3 Population Data Science, Swansea University Medical School, Swansea, Wales, UK; 4 Academic Primary Care, University of Aberdeen School of Medicine and Dentistry, Aberdeen, Scotland, UK; 5 Wellington Faculty of Health, Victoria University of Wellington, Wellington, NZ; 6 Institute of Applied Health Sciences, University of Aberdeen School of Medicine and Dentistry, Aberdeen, Scotland, UK; 7 Strathclyde Institute of Pharmacy and Biomedical Sciences, University of Strathclyde, Glasgow, Scotland, UK; 8 Department of Clinical Pharmacy, College of Pharmacy, Hawler Medical University, Erbil, Iraq; 9 College of Pharmacy, Al-Kitab University, Kirkuk, Iraq; 10 School of Pharmacy, Sefako Makgatho Health Sciences University, Pretoria, South Africa; 11 Department of Mathematics and Statistics, University of Strathclyde, Glasgow, Scotland; 12 Public Health Scotland, Glasgow, Scotland, UK; 13 Centre for Global Health, Usher Institute, The University of Edinburgh, Edinburgh, Scotland, UK; 14 University College Algebra, Zagreb, Croatia

## Abstract

**Background:**

We noted that there remains some confusion in the health-science literature on reporting sample odds ratios as estimated rate ratios in case-control studies.

**Methods:**

We recap historical literature that definitively answered the question of when sample odds ratios (ORs) from a case-control study are consistent estimators for population rate ratios. We use numerical examples to illustrate the magnitude of the disparity between sample ORs in a case-control study and population rate ratios when sufficient conditions for them to be equal are not satisfied.

**Results:**

We stress that in a case-control study, sampling controls from those still at risk at the time of outcome event of the index case is not sufficient for a sample OR to be a consistent estimator for an intelligible rate ratio. In such studies, constancy of the exposure prevalence together with constancy of the hazard ratio (HR) (i.e., the instantaneous rate ratio) over time is sufficient for this result if sampling time is not controlled; if time is controlled, constancy of the HR will suffice. We present numerical examples to illustrate how failure to satisfy these conditions adds a small systematic error to sample ORs as estimates of population rate ratios.

**Conclusions:**

We recommend that researchers understand and critically evaluate all conditions used to interpret their estimates as consistent for a population parameter in case-control studies.

A defining feature of a case-control study design is that selection into the study is intentionally conditional on the occurrence/non-occurrence of an outcome event of interest. A common motivation for this sampling design is to reduce the number of individuals needed in the study so that it can be carried out with less computation or data collection. Traditionally, those experiencing the outcome event are the cases and those not experiencing the outcome event are the controls. Common variations may allow as controls those cases whose outcome event has not occurred by a particular selection time within the study period. This selection time may vary across the controls, a design strategy sometimes called density sampling, or risk-set sampling when the control-selection times are matched to the occurrence times of cases. Although these variations have been studied in depth for decades and described in many textbooks and review articles [1-3], we still encounter reports that include misconceptions about what quantities particular case-control studies estimate.

Some of the authors of the current article (IR, TM, SK, CRS, LR, CR, AS hereafter “the initial authors”) encountered this issue in the review process for a recent paper [4]. In this study, the association between symptomatic coronavirus disease 2019 (COVID-19) and COVID-19 vaccination was estimated using a test-negative case-control design. The study used matched sampling followed by conditional logistic regression. The initial authors believed that risk-set sampling was sufficient for the odds ratio (OR) to be a consistent estimator of a simple, intelligible rate ratio [5-7]. For example, a recent paper stated that “case-cohort and incidence-density sampled case-control studies must report risk ratio and incidence rate ratios, respectively” [5]. This directive turns out to be inaccurate.

In this paper, we seek to discuss and clarify conditions for estimating and reporting of ORs as rate ratios in case-control studies. These conditions were definitively expounded decades ago [1-3]. The OR computed from a case-control study is a mixture of population characteristics with design effects (such as the control sampling strategy) and biases, in addition to the random variation accounted for by conventional statistical methods. This mixture is of no intrinsic scientific or medical interest unless the design effects, biases, and random variation are sufficiently accounted for by the analysis methods. Even with that accounting, however, the population quantity being estimated by a case-control OR will be affected by the sampling design; thus, special attention to the details of this design effect is warranted.

## METHODS

In reference to a specific cohort (fixed set) of individuals, we will use the term “risk” for the average probability of an outcome event over the study period. For more general populations, we will use “rate” for the person-time rate of an outcome over the study period (that is, the expected number of cases over the period divided by the expected person-time at risk of the outcome over the period), and “hazard rate” for its instantaneous (limiting) value at a specific time, see Ch. 3 in [1]. By “consistency of an estimator” for a parameter we will mean that the estimates from a given formula (estimator) tend to cluster ever more tightly around the parameter as the sample size increases.

We first illustrate some basic structure of case-control studies using 2 x 2 tables. In **Table 1** the entry in each cell denotes the number of individuals in a closed cohort for each combination of outcome and exposure categories over a study period. Because it does not affect our discussion, for simplicity we will assume that all cases are observed, and we will ignore random variation in the numbers; hence our points concern underlying population parameters rather than particular sample estimates.

**Table 1:** Number of individuals in a closed cohort for each combination of outcome and exposure categories over a study period

	Exposed	Not exposed
Outcome event	a	b
No outcome event	c	d

The population outcome odds ratio in **Table 1** is *ad/bc*. This is also equal to the population exposure OR.

If we sample controls from those that did not have the outcome event and find that *e* were exposed while *f* were not exposed, then assuming no selection bias (i.e., selection is independent of exposure) *f/e* is a consistent estimator for *d/c*. It follows that the sample odds ratio *af/be* is a consistent estimator of the population OR *ad/bc*.

The exposure odds in the cases relative to the exposure odds in the whole cohort is


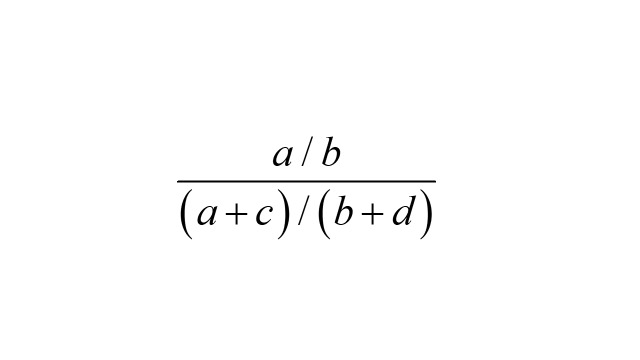
.

However, *a* / (*a* + *c*) is the probability of an outcome event among the exposed, and *b* / (*b* + *d*) is the probability of an outcome event in the unexposed. Therefore, the exposure OR



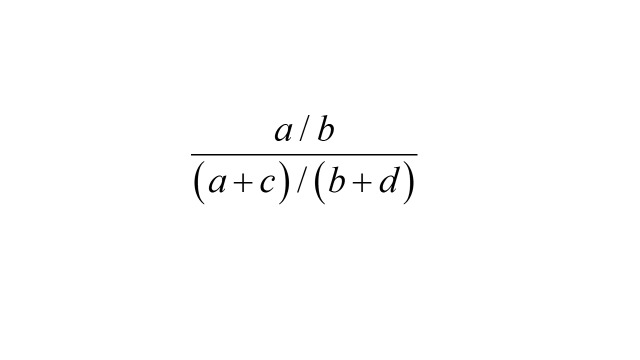



is also the risk ratio



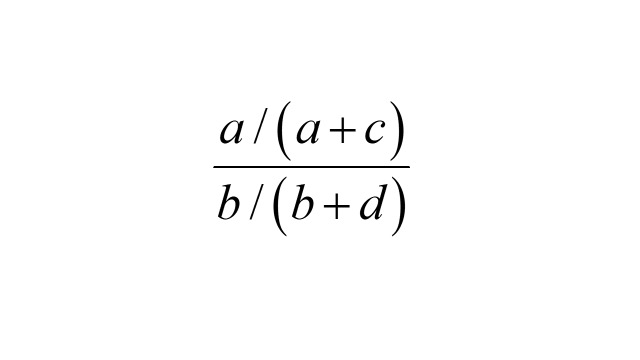



in the population. If we take as controls a sample from the cohort without regard to the outcome event and find g exposed and h not exposed, then, assuming no selection bias,



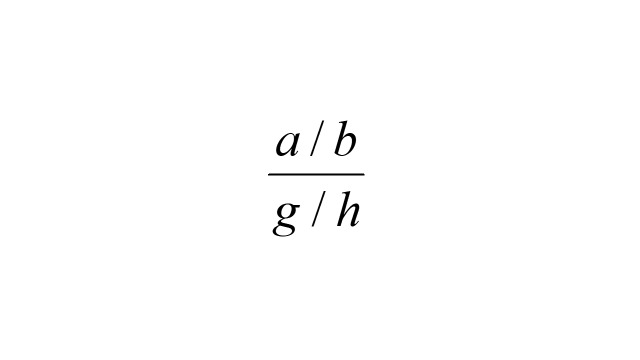



is a consistent estimator for the risk ratio


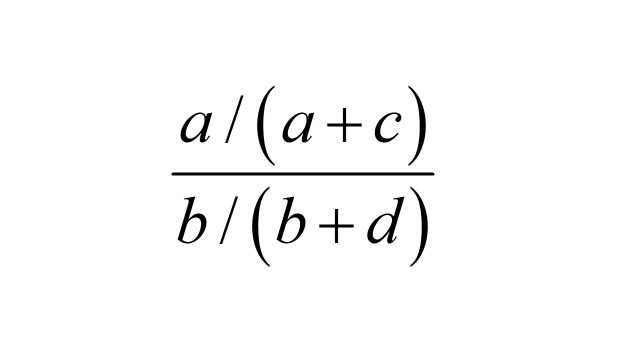
.

It follows that the exposure OR in the cases relative to the cohort sample is a consistent estimator of the cohort risk ratio. Studies that employ this sampling strategy are called case-cohort studies. Note that a person may appear as both a “control” in the cohort sample and as a case in the study (the samples may overlap), which necessitates special formulas for statistical analysis [1].

There are some conditions under which a case-control sample OR is consistent for a population outcome-rate ratio of interest. In particular, this is true if the analysis employs stratification on sampling time of the controls and outcome-event time of the cases, the hazard ratio (HR) is constant over the study period, and there is no selection bias [2]. However, as the strata are made more narrow, more strata will have either no case or no control and thus contribute no information to standard statistical analyses. To ensure that this information loss does not happen, the usual sampling strategy is to match control sampling times to case outcome-event times, i.e., risk-set sampling or matched density sampling [1,2]. If the analysis does not stratify on time, then the additional condition that the population prevalence of exposure is constant over the study period is sufficient for the unmatched odds ratio to also be consistent for the population outcome-rate ratio, and the bias produced by violation of this condition is towards the null (OR = 1) [2].

Note that these statements apply to any method that results in a consistent estimator of OR. For example, in a logistic model, the conclusions follow immediately by noting that the exponential of a coefficient is an OR. The relationship between ORs and rate ratios under risk-set sampling was first noted by Sheehe [8]; see [2] and p. 124-125 of [1] for a review, with extension to logistic regression in [3] and p. 429-435 of [1].

To illustrate the magnitude of the differences between population HRs, population outcome-rate ratios, and the parameters estimated by case-control sample ORs (the OR estimands), we computed these quantities for several examples summarised in **Table 2**. For simplicity, the study period was broken up into intervals over which the outcome event rates for each group and the rates of movement of individuals from the unexposed to exposed group were taken to be constant, and individuals were removed from risk when the outcome occurred (as would be the case with death, for example). The total population is taken to be a closed cohort [1] but with internal migration from the unexposed to exposed subpopulation, as would usually occur with a vaccination program or an infectious agent. In our examples this means that the proportion exposed will increase over the study interval unless the new-exposure rate is zero and exposure increases the hazard rate, and that population ORs and risk ratios would require life tables to compute, hence are omitted here.

**Table 2:** Examples of differences among population rate ratios and the odds-ratio estimands in case-control studies*

Hazard ratio in each interval	Rate of new exposure in each interval	Outcome-rate ratio	Matched odds ratio estimand	Unmatched odds ratio estimand	Final proportion unexposed
0.4, 0.4, 0.4	0, 0, 0	0.40	0.40	0.40	0.79
0.4, 0.4, 0.4	0.1, 0.5, 0.1	0.40	0.40	0.43	0.39
0.4, 0.4, 0.4	0.1, 0.1, 0.1	0.40	0.40	0.41	0.58
2.5, 2.5, 2.5	0, 0, 0	2.50	2.50	2.50	0.82
2.5, 2.5, 2.5	0.1, 0.5, 0.1	2.50	2.50	2.31	0.42
2.5, 2.5, 2.5	0.1, 0.1, 0.1	2.50	2.50	2.46	0.61
0.8. 0.4, 0.2	0, 0, 0	0.47	0.47	0.47	0.79
0.8. 0.4, 0.2	0.1, 0.5, 0.1	0.39	0.44	0.45	0.39
0.8. 0.4, 0.2	0.1, 0.1, 0.1	0.42	0.44	0.44	0.58
1.25, 2.5, 5.0	0, 0, 0	2.83	2.84	2.85	0.82
1.25, 2.5, 5.0	0.1, 0.5, 0.1	3.34	3.02	2.61	0.43
1.25, 2.5, 5.0	0.1, 0.1, 0.1	3.11	3.01	2.88	0.62

*In all examples, intervals are one week each and the rates are per person-week; the initial proportion unexposed is 0.8; the outcome-event rate in the unexposed is 0.025 cases per person-week; and individuals are removed from risk when the outcome occurs.

## RESULTS

Each of the examples in **Table 2** shows calculations with three week-long time intervals where the rate ratio and exposure rate are constant in each week. So, for example, HR = 0.8, 0.4, 0.2 means the ratio of number of outcome events per person time in the exposed relative to the unexposed was 0.8 in the first week, 0.4 in the second week, and 0.2 in the third week. The exposure rate is the rate per person-week at which unexposed individuals become exposed. The rate ratios and ORs were calculated using numeric integration based on 1000 equal-sized subintervals per week, which may lead to slight discrepancies of the ratios presented from theoretical expectations. Further details of the calculation can be found in **Box 1**. R code for the calculator is available on GitHub at https://github.com/EAVE-II/odds-rate-ratio-calculator and this can be used to further explore the sensitivity of this calculation.

Box 1 **Details of numerical calculations**

This discussion is taken from [2]. Let *A_i_* (*t*), *B_i_* (*t*) denote the number of outcome cases and controls sampled respectively by time *t*, with *i* = 1 referring to exposed and *i* = 0 referring to unexposed. Then the unmatched odds ratio is *OR_u_*(*t*) = *A*_1_ (*t*) *B*_0_ (*t*) / *A*_0_ (t) *B*_1_ (*t*).

Let *N* (*t*) denote the size of the total population at risk at time *t*, *P_i_* (*t*), the proportion of *N* (*t*) in exposure group *i* at time *t*, and *R_i_* (*t*) the hazard rate for the outcome event in exposure group *i* at time *t*. In the interval (*t, t* + *dt*) the expected number of cases in exposure group *i* is *a_i_* (*t*) *dt* = *N* (*t*) *P_i_* (*t*) *R_i_* (*t*) *dt* and

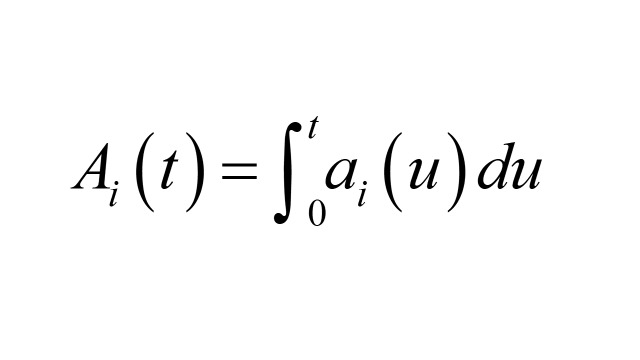
.

Similarly, the expected number of controls in the interval (*t, t* + *dt*) in exposure group *i* is *b_i_* (*t*) *dt* = *a*_+_ (*t*) *P_i_* (*t*) *dt*, where *a*_+_ (*t*) = *a*_0_ (*t*) + *a*_1_ (*t*) and

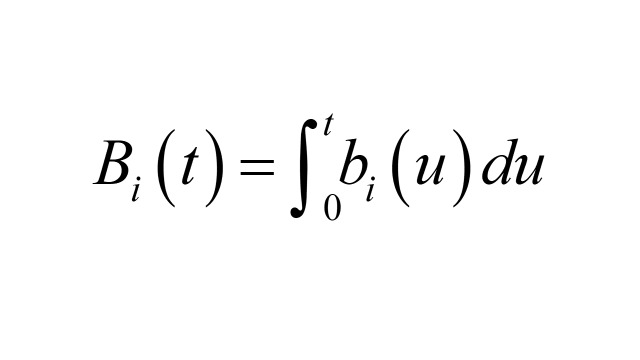
.

The matched OR conditions on membership in each case-control stratum, resulting in the following form

As can be seen in **Table 2**, the HR, rate ratio, matched OR, and unmatched OR estimands are not equal except in some special cases. The magnitudes of the differences are determined by the sizes of the departures from a constant HR (the proportional-hazards assumption) and a constant exposure prevalence (the stable-population assumption). Although the departures used are large and the differences that result are not dramatic, the examples do show that the ratios are not identical without further assumptions. It may be seen that, even if the HR is changing, when the exposure prevalence changes little, both ORs equal or approximate the rate ratio. On the other hand, if the exposure prevalence changes and the HR is constant, only the matched OR equals the rate ratio. In particular, if the exposure prevalence and the HR vary much over the sampling period, the time-matched ORs obtained upon case-control density sampling need not closely approximate the ordinary person-time rate ratio in the source population.

## DISCUSSION

The established convention in statistical studies in epidemiology is to report parameter estimates assuming, but not explicitly spelling out, a set of conditions that are specific to the fitted model and sufficient for the estimator (the formula providing the estimates) to be consistent. These assumptions are usually omitted from research articles because they are standard, and because this allows greater brevity and simplicity in presentation. Nonetheless, we believe that any additional assumptions beyond these that are relied upon by the authors to interpret their estimator as consistent for some population parameter should be carefully evaluated and reported. This kind of discussion is usually expected and often seen for basic validity assumptions such as no confounding, no selection bias, and no measurement error. We advise mention of additional assumptions that concern the mapping of the sample estimates (such as ORs) to targeted population quantities (such as rate ratios).

An additional source of confusion is the use of terms such as “case-control study” and “estimator” without giving the precise definition being used. In A Dictionary of Epidemiology by Porta M, case-control study is defined as:

*The observational epidemiological study of persons with the disease (or another outcome variable) of interest and a suitable control group of persons without the disease (comparison group, reference group). The potential relationship of a suspected risk factor or an attribute to the disease is examined by comparing the diseased and non-diseased subjects with regard to how frequently the factor or attribute is present (or, if quantitative, the levels of the attribute) in each of the groups (diseased and non-diseased)* [9].

We think this definition is somewhat ambiguous as to whether the dependent variable in a case-control study must be exposure rather than the outcome of interest. By contrast, Rothman KJ et al. [1] defined case-control studies as follows:

*Case-control studies are best understood and conducted by defining a source population at the outset, which represents a hypothetical study population in which a cohort study might have been conducted, and by identifying a single disease of interest. If a cohort study were undertaken, the primary tasks would be to identify the exposed and unexposed denominator experience, measured in person-time units of experience or as the number of people in each study cohort, and then to identify the number of cases occurring in each person-time category or study cohort. In a case-control study, these same cases are identified and their exposure status is determined just as in a cohort study, but denominators from which rates could be calculated are not measured. Instead, a control group of study subjects is sampled from the entire source population that gave rise to the cases.*

This definition is in accordance with the long-standard use of disease as the dependent variable in regression models for case-control studies, which is based on extensive theoretical results from the 1970s [1,3]. The idea that it is not possible to estimate outcome ORs in a case-control study and that only exposure-ORs can be estimated, may have contributed to the misplaced claim in [5] that all case-control studies with risk-set sampling must report rate ratios rather than ORs.

In most technical literature, an estimator is called consistent for a parameter if it converges in probability to the parameter; it thus refers to an approximation whose error shrinks toward zero as the sample size becomes large without bound. This type of approximation is quite different from the approximations discussed in the literature relating risk ratios, rate ratios and ORs under “rare-disease assumptions” [1,6]. It appears that in the latter literature, “estimator” is sometimes used to mean “consistent estimator”, and this has generated confusion over when a rare-disease assumption is needed. For example, given an event with positive risk probability (p), the odds *p/*(*1 - p*) of the event may be said to approximate *p* when *p* is “small”, but it is not a consistent estimator of *p* because the approximation error remains positive no matter how large the sample becomes. The same caution applies when using population ORs as approximations to risk ratios; furthermore, as the risk *p* becomes larger, odds and their ratios are increasingly affected by “noncollapsibility” artefacts that do not afflict risks and their ratios [10]. Similar comments apply to use of rate ratios to approximate risk ratios, although the approximation error and artefacts are smaller than that for population ORs [2]. We thus advise authors to be precise about the approximations they are using when making assertions about what they are estimating.

Finally, we have ignored random variation and focused only on large-sample properties. In practice however it will be essential to take account of that variation and also to be on guard against misinterpretations of the statistics [11,12] as well as the small-sample and sparse-data biases that can afflict sample ORs, regardless of what they are estimating [13].

## CONCLUSIONS

Risk-set sampling is not sufficient for a sample OR from a case-control study to be consistent for an intelligible outcome-rate ratio. As has long been known, if the HR is constant over the study period, the case-control sampling is time-matched, the matching is accounted for in the estimator, and there is no source of uncontrolled bias, then consistency follows. If the constant HR condition is dropped, the sample ORs are consistent for a weighted mean of HRs, which under certain conditions may coincide with a population-standardised rate-ratio but otherwise lacks a simple and useful interpretation. In a case-control study that is not matched, the matching assumptions can be replaced by the condition that the ratio of individuals in exposed vs. unexposed groups is constant over time. Generalisation to case-control logistic regression model follows from noting that the exponential of a treatment coefficient in the model equals an OR defined within a sampling process. Consistency for a population quantity follows if there is no source of large-sample bias. Nonetheless, further assumptions beyond the usual validity conditions are needed to equate this OR with a rate ratio [1,2]. In particular, we suggest that if researchers use constancy of the hazard rate or proportion exposed to interpret an OR as a rate ratio, then these additional conditions should be explicitly reported.


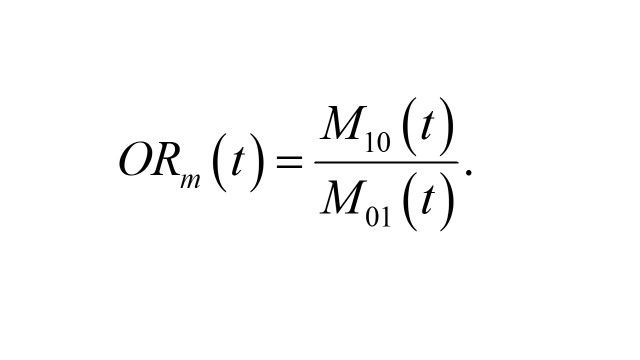


Here,



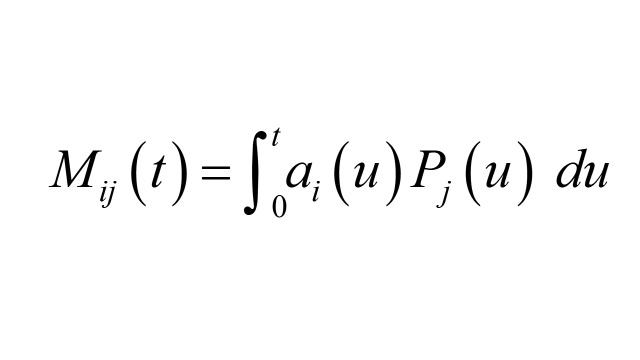



is the expected number of matched pairs over the interval (*t, t* + *dt*) where the case is in exposure group *i*, and the control is in exposure group *j*. Our numerical examples calculate Riemann sum approximations to the above integrals. So, for example, *A_i_* (*t*) is approximated by *Σ_u_ N* (*u*) *P_i_* (*u*) *R_i_* (*u*) *Δu*, where the sum is over time subintervals of length Δ*u*, and *u* is time at the start of each subinterval. *N* (*t*) *P_i_* (*t*) changes discontinuously at the end of each subinterval of length Δ*t* according to

*N* (*t* + Δ*t*) *P*_0_ (t + Δ*t*) = (1- *R*_0_ (*t*) Δ*t* - E (*t*) Δ*t*) *N* (*t*) *P*_0_ (*t*)

*N* (*t* + Δ*t*) *P_1_* (*t* + Δ*t*) = (1 - *R*_1_ (*t*) Δ*t* + E (*t*) Δ*t*) *N* (*t*) *P*_1_ (*t*).

Here, E (*t*) is the rate per subinterval of person time at which unexposed individuals become exposed.
